# Bruxism in a Child with Trisomy 21 (Down Syndrome)—Case Report

**DOI:** 10.3390/jcm13133679

**Published:** 2024-06-24

**Authors:** Lena Sobiech, Izabela Dąbkowska, Wojciech Bekiesz, Karolina Turżańska, Tomasz Blicharski, Katarzyna Sarna-Boś

**Affiliations:** 1 Department of Rehabilitation and Orthopedics, Medical University of Lublin, Jaczewskiego 8, 20-954 Lublin, Poland; lenasobiech@gmail.com (L.S.); tomasz.blicharski@umlub.pl (T.B.); 2 Department of Sports Medicine, Faculty of Health Science, Medical University of Lublin, Chodźki 15, 20-093 Lublin, Poland; 3 Department of Dental Prosthetics, Medical University of Lublin, Chodźki 6, 20-093 Lublin, Poland; katarzyna.sarna-bos@umlub.pl

**Keywords:** bruxism, Down syndrome, 21 trisomy, temporomandibular disorders, tooth wear, case report

## Abstract

Bruxism has been defined in recent years and analyzed in detail as the repetitive activity of the muscles of the masticatory system. Both adults and children experience two different forms of bruxism: daytime and nighttime bruxism. According to the WHO, bruxism affects 5% to 50% of the world’s pediatric population. The symptoms of this disease include tooth wear and fractures, temporomandibular disorders, headaches, behavioral and sleep disorders, and parafunctional habits such as nail biting. According to scientists, psychosocial factors are the most likely factors causing bruxism in children. To this day, we do not have established standards of treatment for children, especially for those with disabilities. The issue of bruxism in children with Down syndrome (DS) is still unexplained. Anatomical abnormalities in the facial skeleton, reduced muscle tone, personality traits, and sleep problems in these people may cause the symptoms of bruxism. Our study aimed to present a clinical case of a 13-year-old girl with DS and symptoms of bruxism. Diagnostics and dental examination in children with intellectual disabilities and a lack of understanding of the disease create a great challenge for the treatment team, including the dentist, physiotherapist, psychologist, and family. Creating standards for treating and preventing bruxism symptoms is demanding, among other things, due to the lack of sufficient scientific research.

## 1. Introduction

### 1.1. Trisomy 21: Presentation and Epidemiology 

Trisomy 21, caused by a complete or partial extra copy of human chromosome 21, is the most commonly diagnosed chromosome disorder in newborns. The incidence is 1:791 live births [1,2,3,4]. The WHO estimates that there are approximately 8 million people with Down syndrome (DS) in the world; it occurs in all ethnic groups and is not gender-dependent [5]. Patients with this disease have reduced cognitive abilities that range from mild to moderate intellectual disability. A delay in psychomotor development is also observed, which means that children with DS need more time to acquire skills that their peers acquire faster [6,7]. Down syndrome is associated with dysmorphic features and several abnormalities in the structure and functioning of the entire body. Characteristic clinical symptoms are abnormalities in the facial skeleton and oral cavity; people affected by DS struggle with the problems of microcephaly, small structures of the nose and teeth, small jaw bones, an open bite, and a protruding and overgrown tongue [7]. Due to the specific structure of the facial skeleton, DS has a disturbed respiratory tract—mouth breathing (MMO). Moreover, stomatognathic problems such as periodontal disease, hypodontia or oligodontia, hypoplasia and atrophy of the main salivary glands, and temporomandibular disorders (TMDs) are more common than in the general population of children [8]. Children with DS have a high rate of dental caries and gaps and filling defects. Additionally, there is a class I malocclusion and poor oral hygiene. Malocclusions in children with Down syndrome, classified as overbites, occur in approximately two-thirds of patients, while a reverse horizontal overbite is observed in approximately 70% of cases. A hypoplastic maxilla or mandible is considered the leading cause of anterior defects. Additionally, there is a hypotonic and furrowed tongue with standard or increased dimensions protruding outside the oral cavity. This structure of the facial skeleton and oral cavity may lead to alveolar dental disorders, resulting in an open bite. Another malocclusion in children with Down syndrome is a crossbite [9]. It is associated with disturbances in the transverse growth of the jaw and disproportions in the width of the dental arches caused by the widening of the lower arch. Due to communication problems and mental impairment, children with DS require constant supervision when brushing their teeth independently [8].

Phenotypic features of this disease also include congenital heart disease, congenital defects and impaired functions of the gastrointestinal tract, hypothyroidism, instability of the glans and gyral vertebrae, reduced auditory and sensory function, immune system deficiency, and muscle hypotonia [4].

### 1.2. Trisomy 21: Treatment

As trisomy 21 is an incurable genetic defect, the treatment used is symptomatic, individually tailored to the problems faced by a given patient, and used to improve the patient’s quality of life. A child with trisomy 21 should be provided with interdisciplinary care and rehabilitation from the first weeks of life [10]. The activities of the therapeutic team should be based primarily on prevention and continuous monitoring of the health condition by several specialists to minimize the risk of consequences of primary diseases [11].

The most common heart defects in people with trisomy 21 are an atypical atrioventricular canal, a hole in the interventricular and/or interatrial septum, and valve regurgitation, usually treated surgically. The detection of valve regurgitation requires prophylaxis against bacterial endocarditis, both before and after the repair of congenital heart disease [12].

Sleep apnea associated with obesity, muscle hypotonia, and other structural abnormalities may cause hypertension, heart disease, stroke, diabetes, and obesity, as well as mental disorders such as depression and paranoia. Therefore, it is crucial to eliminate this problem. The most commonly used method is positive airway pressure therapy, but the patient may not tolerate it [13].

Conductive and sensorineural hearing loss occurs in up to 70% of people with Down syndrome. Poor hearing causes problems with speech, which is the essential means of communication; in such a case, it is crucial to equip the patient with a hearing aid and provide speech therapy care. Studies have been conducted on the use of the “Talk It” application in therapy for augmentative and alternative communication in people with Down syndrome, which showed a significant improvement in language skills, particularly for naming [14].

Rehabilitation plays a huge role in becoming independent and improving the quality of life of such patients. Physiotherapy interventions based on resistance training are effective in improving the strength of the upper and lower limbs, and interventions based on core stabilization exercises, treadmill training, and vibration therapy have a positive effect on balance, particularly in reducing mediolateral displacements of the center of gravity [2]. Hand therapy, which improves the patient’s manual skills, provides people with Down syndrome the ability to independently cope with everyday activities [15].

It is essential to combine exercises that positively affect the patient’s fitness with artistic elements that influence the mental and emotional sphere, which is as important as the physical one. Thanks to the interdisciplinary care of a team of specialists, a patient with Down syndrome becomes an independent individual who functions well in society and enjoys a good and long life [16].

### 1.3. Bruxism: Presentation and Epidemiology 

According to the World Health Organization (WHO), bruxism is the unconscious, habitual clenching and grinding of teeth and/or supporting and pushing of the jaw. Bruxism affects 5 to 50% of children, including disabled children [17,18]. This large discrepancy is due to the different ages of the studied children and ethnic and environmental differences. Taking into account the time of occurrence of bruxism, we distinguish night bruxism SB (sleep bruxism) and day bruxism AB (awake bruxism) [17,18,19]. SB is characterized by increased muscle activity during sleep and is described as rhythmic (phasic, where muscles alternately tense and relax) and non-rhythmic (tonic, where muscles tense and do not relax for some time). Daytime bruxism, in turn, is characterized by increased muscle activity while awake and after waking up. Symptoms may include intermittent or continuous tooth contact and/or rapid, sudden movement of the jaw (thrusting) or habitual holding of the jaw in one position (bracing). Bruxism often has a psychological basis. Long-term stress and psychological trauma may contribute to increased tension of the jaw muscles, especially the masseter muscles, which leads to unconscious teeth clenching [16]. According to the International Classification of Sleep Disorders, the minimum criteria for the diagnosis of sleep bruxism are the presence of frequent or regular (at least three nights a week for at least three months) teeth-grinding sounds during sleep and one of the following symptoms: abnormal tooth wear, transient morning fatigue or pain jaw muscles, transient headaches, or morning blockage of the temporomandibular joints [20]. This is a widespread phenomenon in children, especially in those with problems falling asleep and staying asleep.

### 1.4. Bruxism: Treatment 

There are many therapeutic methods to treat bruxism. For choosing the most proper method, it is necessary to distinguish whether a person suffers from daytime AB (awake) bruxism or nighttime (sleep bruxism SB). When the problem occurs at night, relaxation splints are helpful in conservative treatment as they relieve the muscles and temporomandibular joints and protect the teeth against pathological wear of their occlusal surfaces [20]. Physiotherapy also brings relief by restoring the balance of muscle tension in the face and neck area and the range of jaw mobility. Muscle relaxation techniques and fascial massages are used [21]. 

When choosing a method, looking at the cause of bruxism is crucial. If it is psychological, psychotherapy should be considered. Another method of treating bruxism is the use of botulinum toxin, a neurotoxin produced by the Gram-positive bacterium Clostridium botulinum. Botulinum toxin type A (BTX-A) inhibits the functioning of skeletal muscles, hindering the production of acetylcholine and inactivating calcium channels in nerve endings [22]. Intramuscular injections can reduce the frequency of bruxism episodes and the level of pain resulting from these parafunctions. Another method of treating bruxism that is still being tested is pharmacology. The trials include muscle relaxants such as tolperisone and benzodiazepines, antidepressants, anxiolytics, anticonvulsants, and beta-blockers [23]. However, the use of drugs for an extended period is associated with the risk of side effects and addiction [24]. Currently, no single algorithm for treating bruxism exists; all of the methods mentioned above focus on treating symptoms and preventing further complications [23]. This study aims to present a clinical case of a 13-year-old girl with DS and symptoms of bruxism.

## 2. Case Report

The case report was written in accordance with the CARE case report guidelines.

### 2.1. Patient 

In December 2023, a 13-year-old girl with Down syndrome visited a dentist for treatment of temporomandibular joint disorders and symptoms of worn teeth. According to the patient’s medical history, she was being treated for hypothyroidism by an endocrinologist and was permanently taking Euthyrox (Levothyroxine) at a dose of 75 mg/day. Due to the difficulty in self-assessing the girl’s symptoms, all information was obtained from the child’s parents. From an interview with the patient’s mother, in the years 2018–2020, the patient was treated by a psychiatrist for anxiety, and during this period, she took Seronil (Fluoxetin) at a dose of 10 mg ¼ tablet once per day. Moreover, the child was healthy and did not complain of other health problems. Regarding the social area, the child studied in a school for special educational needs and disabled children. She established and maintained contact with peers, communicated verbally, expressed her needs, shared comments, and conducted alternating dialogs.

### 2.2. Patient Examination 

**Figure 1:**
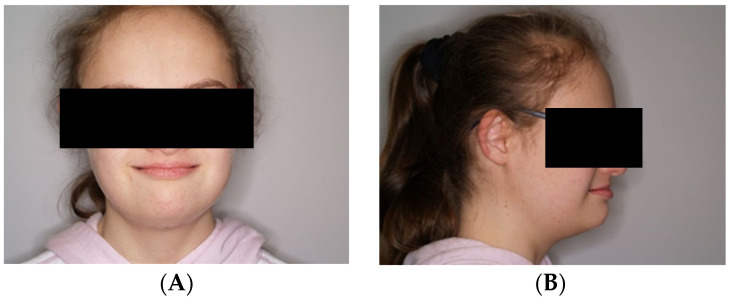
(**A**,**B**) Resting position of the lower jaw.

**Figure 2:**
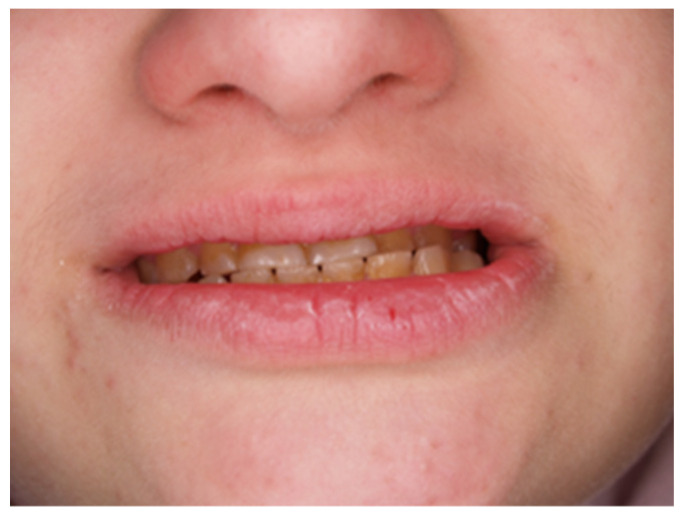
Photo with closed mouth. Oblique space, occlusal plane, and left-sided crossbite.

**Figure 3:**
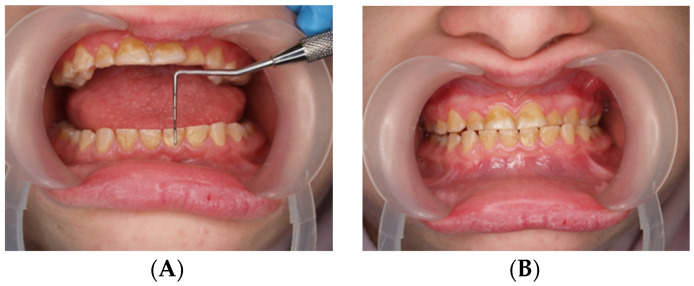
(**A**,**B**) Assessment of the height of worn teeth.

During the first adaptation visit, the patient underwent a clinical palpation examination to assess the height of the mandibular occlusion and severity of tooth wear, the range of jaw abduction, and hypermobility in the temporomandibular joint. There was no limitation of jaw mobility (positive three-finger test). The resting position of the lower jaw is shown in Figure 1. However, a left-sided lateral crossbite was found (Figure 2) and pathological tooth wear in the range of 1/4–1/3 of the height of the clinical crown was found (Figure 3). The intraoral examination assessed the angle class, cuspid class, vertical and horizontal overbite, and incisor protrusion/retrusion. Tongue dysfunction and tension of facial and masticatory muscles were also assessed. The patient was diagnosed with a skeletal class III malocclusion and had an oral hygiene index OHI 6, bleeding on probing BoP 40%, and approximal plaque index API 50%.

Due to the child’s high degree of anxiety and fear of an X-ray examination, as well as unplanned orthodontic treatment, there were no indications to take X-rays (panoramic and cephalometric imaging).

The parents informed the medical team about the sounds of teeth grinding often heard during the patient’s sleep and during classes—information provided to parents by teachers.

Additionally, sleep disorders were assessed using the Children’s Sleep Habits Questionnaire (CSHQ) (Table 1) [25,26]. Due to very rare episodes of sleep apnea (twice in a lifetime—Table 1), further diagnostics in this direction were decided not necessary.

The CQHS test score is calculated as the sum of answers to all point questions, ranging from 33 to 99. The answers to each question were determined according to the frequency of the problem; depending on the assignment to one of the three ranges, one, two, or three points were awarded. The score increases according to the intensity of the negative trait; one point is assigned if the problem is rare, and three points are assigned if it is frequent. A questionnaire result of over 41 points indicates sleep disorders in children, as this cutoff has been shown to accurately identify 80% of children with clinically diagnosed sleep disorders [25,26].

### 2.3. Dentistry and Rehabilitation Treatment

During the next dental visit, the patient and parents were instructed in daily hygiene and myofunctional therapy of the masseter and temporal muscles. The myofunctional therapy was aimed at improving the respiratory path (the patient breathes through the mouth) and the incorrect resting position of the tongue, which consequently affects malocclusion and bruxism. In addition, to improve the position of the lower jaw and close the mouth, the mother was asked to perform trigger point therapy for the lateral pterygoid, masseter, and temporal muscles, as well as intraoral therapy 2–3 times a week. Treatment of muscle tension with botulinum toxin was abandoned due to generalized hypotonia (floppy child). 

The dentist also sealed the fissures of the premolars and molars to protect the teeth against caries. The sealing treatment should be repeated in about 2–3 years. 

Another follow-up visit took place after approximately three months to assess the muscle tone in the facial area and the state of oral hygiene. 

### 2.4. Follow-Up Outcomes

During the next follow-up visit, it was found that the fissure sealant remained on the chewing surfaces of only 4 of the 16 teeth after three months. However, a significant improvement in oral hygiene was observed. Longer-lasting mouth closure and improvement in the lower jaw position were also observed.

In the upcoming phase of treatment, the dental team plans to construct an acrylic relaxation splint in a dental laboratory after making impressions of the patient’s dental arches using impression material. This splint is designed to elevate the bite and prevent potential growth restriction at the base of the jawbone. The estimated duration of use of the relaxation splint is, at most, three months. The splint will only be used during sleep, ensuring the patient’s comfort and compliance with the treatment plan.

Due to the specific structure of the facial skeleton, therapy results may need improvement. Moreover, the short follow-up period does not allow for a reliable and complete assessment of the outcome and treatment progress, but the results seem satisfactory. 

## 3. Discussion

Symptoms of bruxism, such as voluntary teeth grinding or clenching and/or bracing, jaw thrusting during sleep, abnormal tooth wear, transient morning fatigue, jaw muscle pain, transient headache, or morning blockage of the temporomandibular joints may be intensified in a child with intellectual and motor disabilities, such as a person with Down syndrome (DS). Attention should be paid to studies showing that people with DS have difficulties expressing and reporting pain and assessing the strength of touch and degree of temperature [27,28]. Due to the lack of understanding of the problem and situation during a dental examination of a disabled person, making a diagnosis is complex and requires more extended patient observation. According to the Children’s Sleep Habits Questionnaire (CSHQ), our patient exhibited the above symptoms, i.e., excessive tooth wear and night grinding (Table 1). Moreover, the dental examination did not reveal any tenderness of the masseter muscles. However, the tongue recess was visible. The study by Dicieri-Pereira et al. investigated orofacial pain in people with Down syndrome and determined the relationship with masticatory disorders. It has been found that men with Down syndrome more often suffer from myofascial pain, muscle hypotonia, obstructive sleep apnea, and snoring. Women with DS, on the other hand, report joint pain and SB more often [29]. The hypermobility characteristic of Down syndrome also confirms the problem with disorders of the temporomandibular joints and their excessive mobility, which is manifested by cracking in the joint and malocclusion [7].

Researchers also suggest expanding the diagnosis of bruxism, not only based on an interview conducted with the child and caregivers along with the clinical symptoms, but also through assessment recording muscle activity electromyography (EMG) and assessment using a polysomnography (PSG) device, which records the patient’s behavior at night [30,31]. Unfortunately, additional diagnostics using EMG or PSG is troublesome, especially in a person with intellectual disability, due to the need to participate with them outside of the home in a clinical setting and due to the high cost of the test. 

The research conducted by Gomes A.A. et al. is of significant importance. They discovered that children with attention deficit hyperactivity disorder and attention deficit disorder are more likely to develop symptoms of bruxism, which manifests as a release of tension during dreams. This theory could potentially be extended to children with Down syndrome [32]. Additionally, children with DS often exhibit repetitive behaviors (stereotypes and motor rituals) more frequently than their healthy counterparts, which could contribute to the development of parafunctions [33].

Determining the clinical management strategy for a patient with symptoms of bruxism is a challenge for the treatment team, i.e., the dentist, pediatrician, and family, especially if it concerns a disabled person. Research has shown that people with Down syndrome often have sleep disorders, which have a significant impact on SB [34]. Sleeping with the mouth open and breathing through the mouth, protruding the mandible in front of the jawbone, is associated with the specific structure of the facial skeleton in these people, which is characterized by a poorly developed middle third of the face and macroglossia (hypertrophy of the tongue) combined with disorders of psychosomatic development, which may facilitate the development of temporomandibular disorders (TMD) and bruxism in people with Down syndrome [35,36,37].

The relationship between SB and the degree of mental disability is debatable. In 2007, the researchers Lopez-Perez et al. found a statistically significant difference between different types of trisomy 21. People with DS and genetic mosaicism are more often exposed to SB [38]. Our patient, diagnosed with trisomy 21, is not in the risk group. 

Most clinicians approach the treatment of bruxism empirically. It is believed that in children between the ages of three and five years, the chewing surfaces of the teeth undergo physiological wear to allow for the growth and development of the jaws. The incidence of bruxism has also been shown to decrease from approximately age 9 to 10 years, supporting the belief that most children with bruxism will not exhibit symptoms of the SB during adolescence and adulthood. Researchers have observed that the symptoms of SB in children with Down syndrome also decrease with age (approximately 12 years) [39].

One of the basic therapies for bruxism is dental treatment involving the use of occlusion devices during sleep. Occlusal splints are designed to protect teeth against pathological abrasion and reduce the activity of facial muscles. The results of splint treatment are satisfactory, and adults willingly use this kind of treatment [40]. The treatment of small children with occlusal appliances is debatable, as they could impair future alveolar bone growth and malocclusion. Therefore, further research is needed to check the effectiveness of occlusal therapy in children with symptoms of bruxism [39,41].

There are few reports in the scientific literature on the treatment of bruxism in children with DS. One of the methods of treating the symptoms of bruxism is physiotherapy. Physiotherapy treatments aim to relax facial muscles and reduce pain. Treatments such as massages, thermotherapy, and acupuncture laser therapy are recommended. For children with DS and bruxism, laser-point stimulation is recommended due to the painless and short duration of the procedure [42]. In a study, Salgueiro et al. showed that acupuncture treatment alleviated the symptoms of craniofacial pain in these children and reduced the level of cortisol secreted in states of anxiety and stress [43].

In the publication by Luconi et al., a review of the literature ranging from 1988 to 2020 on treating bruxism in children with Down Syndrome was undertaken. Only four studies have been conducted on this issue. The researchers Lamma and Cocchi, among others, treated children with DS with pharmacological therapy using drugs from the benzodiazepine and carbamazepine groups, intended to reduce anxiety and stress symptoms. Our patient’s interview contained information about Seronil treatment for two years. Due to no difference in behavior, the parents stopped administering the drug. Due to the lack of information about the teeth during this period, we cannot determine whether the symptoms of bruxism may have increased. In turn, Neil and Jones tried to eliminate negative behaviors using behavioral therapy, strengthening positive behaviors in disabled children [44]. 

Researchers Areias et al. reached interesting conclusions. They found that bruxism is not only about bad habits and their negative consequences but also protects against caries in children with DS by creating smoother occlusal surfaces, enabling self-cleaning with the tongue and facilitating oral cavity hygiene [45]. 

The early diagnosis of bruxism in a child with Down syndrome is essential, not only because of tooth wear or to alleviate craniofacial pain, but also to inhibit craniofacial changes and allow the possible reconstruction of lost structures [46].

## 4. Conclusions

The treatment of people with intellectual disabilities poses considerable difficulties in medical diagnosis, and it is currently difficult to define standards of practice. Currently, there are no guidelines for the treatment of bruxism in healthy children and adolescents, much less in people with disabilities. SB in children with Down syndrome is a significant clinical problem due to its negative consequences, such as tooth wear, facial muscle pain, and facial deformation. Parents are encouraged to take preventive measures against parafunctions and observe their children during sleep. Such measures should eliminate parafunctions and include systematic check-ups by a dentist, including treatments such as varnishing and sealing of healthy teeth, rehabilitation with a relaxation splint, polishing of sharp enamel edges, reconstruction of carious lesions with a composite material with toughness similar to the hardness of the enamel, avoiding ceramic restorations due to rapid abrasion the surfaces of opposing teeth, and performing reconstructions and flat-cusp restorations to eliminate the formation of injury nodes on the occlusal plane. These actions prevent further tooth wear, lower the height of the occlusion, and lower the symptoms of bruxism by reducing muscle tension. The literature results also suggests that good sleep hygiene may help treat SB in children.

As our case report shows, even a relatively short observation period shows the positive effect of this approach.

## Figures and Tables

**Table 1:** Children’s Sleep Habits Questionnaire (CSHQ).

Write in Child’ Bedtime:	Usually 5–7	Some Times 2–4	Rarely 0–1
Child goes to bed at the same time at night	1		
Child falls asleep within 20 min after going to bed		2	
Child falls asleep alone in own bed			3
Child falls asleep in parent’s/sibling’s bed			1
Child needs parent in the room to fall asleep	3		
Child struggles at bedtime (cries, refuses to stay in bed, etc.)			1
Child is afraid of sleeping in the dark		2	
Child is afraid of sleeping alone		2	
**Sleep behavior** Child’s usual amount of sleep each day	Hours: 8 h	Minutes: 30 min	
Child sleeps too little			1
Child sleeps the right amount	1		
Child sleeps about the same amount each day	1		
Child wets the bed at night			1
Child talks during sleep			1
Child is restless and moves a lot during sleep			1
Child sleepwalks during the night			1
Child moves to someone else’s bed during the night (parent, brother, etc.)		2	
Child grinds teeth during sleep (your dentist may have told you this)			1
Child snores loudly		2	
Child seems to stop breathing during sleep			1
Child has trouble sleeping away from home (visiting relatives, vacation)			1
Child awakens during night screaming, sweating, and inconsolable			1
Child awakens, alarmed by a frightening dream			1
**Walking during the night**			
Child awakes once during the night			1
Child awakens more than once during the night			1
**Morning walking** Write the time of day that the child usually wakes in the morning: 6.00 a. m.			
Child wakes up by themselves			3
Child wakes up in negative mood		2	
Adults or siblings wake up child	3		
Child has difficulty getting out of bed in the morning		2	
Child takes a long time to become alert in the morning		2	
**Daytime sleepiness**			
Child seems tired			1
During the past week, your child has appeared very sleepy or has fallen asleep during the following:			1
	Not sleepy	Very sleepy	Falls Asleep
Watching TV	1		
Riding in car	1		
total	49		

## Data Availability

The original contributions presented in the study are included in the article, further inquiries can be directed to the corresponding author.
